# Mucosal-associated invariant T cell alterations during the development of human type 1 diabetes

**DOI:** 10.1007/s00125-020-05257-7

**Published:** 2020-09-03

**Authors:** Ahmad M. Gazali, Anna-Mari Schroderus, Kirsti Näntö-Salonen, Reeta Rintamäki, Jussi Pihlajamäki, Mikael Knip, Riitta Veijola, Jorma Toppari, Jorma Ilonen, Tuure Kinnunen

**Affiliations:** 1 Department of Clinical Microbiology, Institute of Clinical Medicine, University of Eastern Finland, Kuopio, Finland; 2 Present Address: Faculty of Industrial Sciences and Technology, Universiti Malaysia Pahang, Pahang, Malaysia; 3 Department of Pediatrics, Turku University Hospital, Turku, Finland; 4 Department of Medicine, Kuopio University Hospital, Kuopio, Finland; 5 Institute of Public Health and Clinical Nutrition, University of Eastern Finland, Kuopio, Finland; 6 Clinical Nutrition and Obesity Center, Kuopio University Hospital, Kuopio, Finland; 7 Tampere Center for Child Health Research, Tampere University Hospital, Tampere, Finland; 8 Children’s Hospital, University of Helsinki and Helsinki University Hospital, Helsinki, Finland; 9 Research Program for Clinical and Molecular Metabolism, Faculty of Medicine, University of Helsinki, Helsinki, Finland; 10 Folkhälsan Research Center, Helsinki, Finland; 11 PEDEGO Research Unit, Department of Pediatrics, Medical Research Center, Oulu University Hospital and University of Oulu, Oulu, Finland; 12 Institute of Biomedicine, Research Centre for Integrative Physiology and Pharmacology, University of Turku, Turku, Finland; 13 Immunogenetics Laboratory, Institute of Biomedicine, University of Turku, Turku, Finland; 14 Clinical Microbiology, Turku University Hospital, Turku, Finland; 15 Eastern Finland Laboratory Centre (ISLAB), Kuopio, Finland

**Keywords:** Autoimmunity, Human, Immunophenotyping, MAIT cells, Mucosal immunity, T cells, Type 1 diabetes

## Abstract

**Aims/hypothesis:**

Mucosal-associated invariant T (MAIT) cells are innate-like T cells that recognise derivatives of bacterial riboflavin metabolites presented by MHC-Ib-related protein 1 (MR1) molecules and are important effector cells for mucosal immunity. Their development can be influenced by the intestinal microbiome. Since the development of type 1 diabetes has been associated with changes in the gut microbiome, this can be hypothesised to lead to alterations in circulating MAIT cells. Accordingly, peripheral blood MAIT cell alterations have been reported previously in patients with type 1 diabetes. However, a comprehensive analysis of the frequency and phenotype of circulating MAIT cells at different stages of type 1 diabetes progression is currently lacking.

**Methods:**

We analysed the frequency, phenotype and functionality of peripheral blood MAIT cells, as well as γδ T cells, invariant natural killer T (iNKT) cells and natural killer (NK) cells with flow cytometry in a cross-sectional paediatric cohort (aged 2–15) consisting of 51 children with newly diagnosed type 1 diabetes, 27 autoantibody-positive (AAb^+^) at-risk children, and 113 healthy control children of similar age and HLA class II background. The frequency of MAIT cells was also assessed in a separate cross-sectional adult cohort (aged 19–39) of 33 adults with established type 1 diabetes and 37 healthy individuals of similar age.

**Results:**

Children with newly diagnosed type 1 diabetes displayed a proportional increase of CD8^−^CD27^−^ MAIT cells compared with healthy control children (median 4.6% vs 3.1% of MAIT cells, respectively, *p* = 0.004), which was associated with reduced expression of C-C chemokine receptor (CCR)5 (median 90.0% vs 94.3% of MAIT cells, *p* = 0.02) and β7 integrin (median 73.5% vs 81.7% of MAIT cells, *p* = 0.004), as well as decreased production of IFN-γ (median 57.1% vs 69.3% of MAIT cells, *p* = 0.04) by the MAIT cells. The frequency of MAIT cells was also decreased in AAb^+^ children who later progressed to type 1 diabetes compared with healthy control children (median 0.44% vs 0.96% of CD3^+^ T cells, *p* = 0.04), as well as in adult patients with a short duration of type 1 diabetes (less than 6 years after diagnosis) compared with control individuals (median 0.87% vs 2.19% of CD3^+^ T cells, *p* = 0.007). No alterations in γδ T cell, iNKT cell or NK cell frequencies were observed in children with type 1 diabetes or in AAb^+^ children, with the exception of an increased frequency of IL-17A^+^ γδ T cells in children with newly diagnosed diabetes compared with healthy control children (median 1.58% vs 1.09% of γδ T cells, *p* = 0.002).

**Conclusions/interpretation:**

Changes in the frequency and phenotype of circulating MAIT cells were detectable before, at the onset and after diagnosis of type 1 diabetes in cross-sectional cohorts. Our results suggest a possible temporal association between peripheral blood MAIT cell alterations and the clinical onset of type 1 diabetes.

**Figure:**
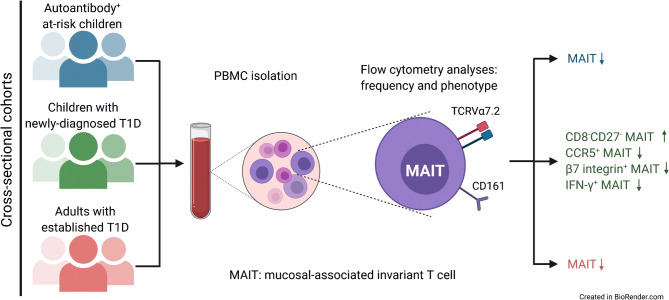
Graphical abstract

**Electronic supplementary material:**

The online version of this article (10.1007/s00125-020-05257-7) contains peer-reviewed but unedited supplementary material, which is available to authorised users.

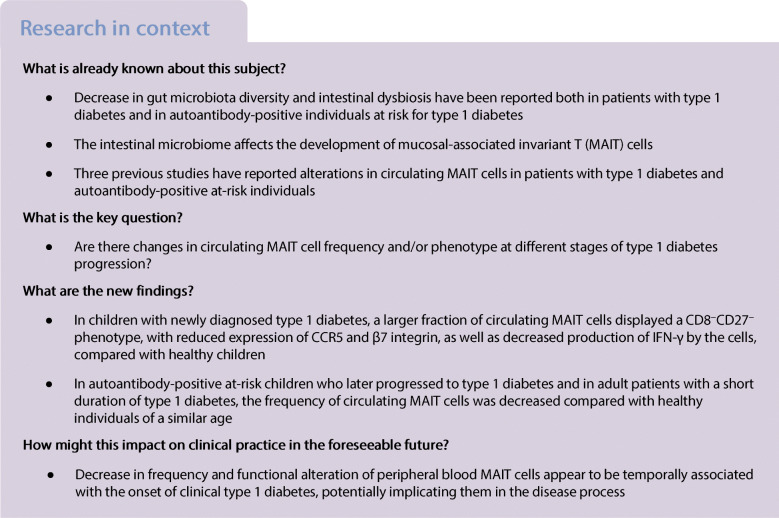



## Introduction

Type 1 diabetes is a chronic autoimmune disease caused by progressive T cell-mediated destruction of insulin-producing beta cells in the pancreas [1]. The clinical presentation of type 1 diabetes is preceded by a period of asymptomatic autoimmunity, during which autoantibodies to islet antigens are almost invariably detected [2, 3].

The incidence of type 1 diabetes has increased considerably in the past 30 years [4, 5]. Despite over 50 susceptibility loci shown to contribute to the development of type 1 diabetes in humans [6, 7], the autoimmune process in genetically at-risk individuals is probably driven by environmental factors, such as infections and diet [8]. Importantly, the composition of the intestinal microbiome has recently been associated with the development of type 1 diabetes. Compared with healthy control individuals of similar age, both patients with type 1 diabetes [9–12] and autoantibody-positive (AAb^+^) children at risk for type 1 diabetes [13–16] have been reported to display decreased bacterial diversity as well as intestinal dysbiosis commonly characterised by increased numbers of *Bacteroidetes* species in the gut microbiome.

The intestinal microbiome also plays a key role in the development of certain subsets of innate-like T cells, such as the mucosal-associated invariant T (MAIT) cells. MAIT cells are preferentially localised in mucosal tissues, including gut, and are largely absent in germ-free mice [17, 18]. Together with γδ T cells and invariant natural killer T (iNKT) cells, MAIT cells are classified as unconventional T cells (UCTs) [19]. MAIT cells express a conserved T cell receptor (TCR) comprising an invariant Vα7.2-Jα33 chain, and they recognise metabolites originating from microbial biosynthesis presented by MHC-Ib-related protein 1 (MR1) on antigen-presenting cells [19]. Upon activation, MAIT cells produce several proinflammatory cytokines, such as IFN-γ and IL-17A, and display cytotoxic effector function against cells infected with certain pathogens [20]. Similar to conventional T cells, MAIT cells develop in the thymus before migrating into the peripheral blood and accumulate in circulation with age [18, 21, 22]. Human peripheral blood MAIT cells express high levels of CD161 and IL-18 receptor α, which together with TCR Vα7.2 can be used in their identification [21].

In recent years, alterations in the circulating MAIT compartment have been observed in multiple autoimmune diseases, such as inflammatory bowel disease (IBD) [23–26], systemic lupus erythematosus (SLE) [27, 28], rheumatoid arthritis [27, 29, 30] and multiple sclerosis [31–33]. The first published study on MAIT cells in patients with type 1 diabetes reported a comparable frequency of circulating CD8^+^CD161^bright^ ‘MAIT-like’ cells in individuals with type 1 diabetes compared with healthy control individuals [34]. A more recent study observed a markedly reduced frequency of circulating MAIT cells in patients with newly diagnosed type 1 diabetes [35]. One more study suggested that the frequency of circulating MAIT cells was also reduced in AAb^+^ at-risk individuals [36]. Variable alterations in CD25, programmed cell death protein 1 (PD-1), C-C chemokine receptor type (CCR)6 and CD27 surface marker expression, as well as IFN-γ and IL-4 production, by peripheral blood MAIT cells from individuals with type 1 diabetes have also been reported in these studies [34, 35].

In order to better understand the role of MAIT cells during type 1 diabetes development, we analysed blood MAIT cell frequency, phenotype and function in samples from individuals at different stages of diabetes progression.

## Methods

### Study participants

The paediatric study cohort comprised a total of 51 children with newly diagnosed type 1 diabetes, 27 AAb^+^ children, and 113 autoantibody-negative healthy children (Table 1). Among the AAb^+^ children, 11 were diagnosed with type 1 diabetes 3–33 months (mean ± SD 13.7 ± 10.5 months) after sampling (progressors) and 16 had not progressed to clinical disease (non-progressors) during the mean 3 year follow-up after sampling. Except for children with newly diagnosed type 1 diabetes, all study participants, including the autoantibody-negative healthy control children, participated in the Finnish Type 1 Diabetes Prediction and Prevention (DIPP) follow-up study and had HLA genotypes associated with increased risk for type 1 diabetes [37]. Autoantibody-positivity was analysed in the children at sampling, as previously described [2]. AAb^+^ children were positive for two or more biochemical autoantibodies (insulin autoantibodies [IAA], insulinoma-associated-2 antibodies [IA-2A], GAD antibodies [GADA] and/or zinc transporter 8 autoantibodies [ZnT8A]).

**Table 1:** Characteristics of study participants

Participant group	*n* (male/female)	Age (years) (mean ± SD)	Range (years)	T1D duration (mean ± SD)	Range
Paediatric T1D	51 (25/26)	8.6 ± 3.9	2–15	<1 week	N/A
Paediatric AAb^+^	27 (14/13)	8.6 ± 4.5	2–15	N/A	N/A
Progressors	11 (6/5)	7.7 ± 4.5	2–13	N/A	N/A
Nonprogressors	16 (8/8)	9.0 ± 4.5	2–15	N/A	N/A
Paediatric control	113 (64/49)	8.9 ± 3.9	2–15	N/A	N/A
Adult T1D	33 (19/14)	27.1 ± 6.4	19–39	11.9 ± 8.0 y	0.4–30 y
Adult control	37 (14/23)	26.2 ± 4.4	20–38	N/A	N/A

N/A, not applicable; T1D, type 1 diabetes; y, years

The adult study cohort comprised 33 adults with established type 1 diabetes and 37 healthy individuals (Table 1).

The study was approved by local ethics committees in the participating university hospitals. All individuals participating in the study and/or their legal guardians provided written informed consent, as mandated by the Declaration of Helsinki.

### PBMC sample preparation

Peripheral blood mononuclear cells (PBMCs) were isolated from peripheral blood samples as previously described [38]. Fresh PBMCs from paediatric participants and frozen PBMCs (cryopreserved in 10% DMSO) from adult participants were used for cell culture and immunostaining experiments.

### Cell culture and stimulation

PBMCs were seeded in U-bottom 96-well plates (10^6^ per 200 μl per well) and cultured for 16–18 h (37°C, 5% CO_2_) in RPMI 1640 complete medium with 5% human AB serum. Cells were either left unstimulated or stimulated with whole *Escherichia coli* bacteria (ATCC strain 25922, Manassas, VA, USA) fixed with 1% paraformaldehyde for 5 min [39], or with a combination of IL-12 and IL-18 (both at 50 ng/ml, Peprotech, Cranbury, NJ, USA). Some samples were preincubated either with anti-MR1 blocking antibody (20 μg/ml, clone 26.5, BioLegend, San Diego, CA, USA) or with IgG2a isotype control (20 μg/ml, clone MPOC-173, BioLegend) prior to *E.coli* stimulation.

### Flow-cytometric analyses

Viability staining was performed on PBMCs using Zombie Aqua dye (BioLegend) according to the manufacturer’s instructions. Immunostaining for surface markers was subsequently performed on at least 10^6^ PBMCs per staining by incubating the cells with a panel of fluorochrome-labelled antibodies (ESM Table 1) for 20–30 min. For cytokine analyses PBMCs were stimulated for 5 h with 20 ng/ml phorbol myristic acid (PMA; Sigma Aldrich, St Louis, MO, USA), 500 ng/ml ionomycin (Sigma Aldrich), and 3 μg/ml brefeldin A (eBioscience, San Diego, CA, USA). Fixation and permeabilisation were performed using the Intracellular Fixation and Permeabilization Buffer set (eBioscience), followed by staining for intracellular cytokines (ESM Table 1) for 30 min. The samples were acquired on FACSCanto II (BD Biosciences, San Jose, CA, USA) or Cytoflex S (Beckman Coulter, Indianapolis, IN, USA) flow cytometers (ESM Table 1). Coded samples were used throughout, and flow-cytometric data was analysed by FlowJo v10.4.2 (BD) blinded to the clinical classification of the sample.

### Statistical analyses

Statistical analyses were performed using Prism software version 7.05 (GraphPad Software, San Diego, CA, USA). Mann–Whitney *U* test or Kruskal–Wallis test with Dunn’s multiple comparison post hoc test were used for statistical comparisons. No power calculations were made as the study was exploratory in nature with very limited previous data to support the calculations, and sample sizes were determined in part by feasibility. Age as a covariate was addressed by comparing the slopes and/or elevations of linear regression lines. Relationships between different variables were examined using Spearman correlation coefficient. To increase statistical power, samples from different study groups were pooled for correlation analyses in cases where the slopes of the linear regression lines did not differ statistically between the study groups. A *p* value <0.05 was considered to indicate statistical significance.

## Results

### Decreased frequency of MAIT cells in AAb^+^ children who later progressed to type 1 diabetes and in adult patients a short time after onset of diabetes

**Fig. 1:**
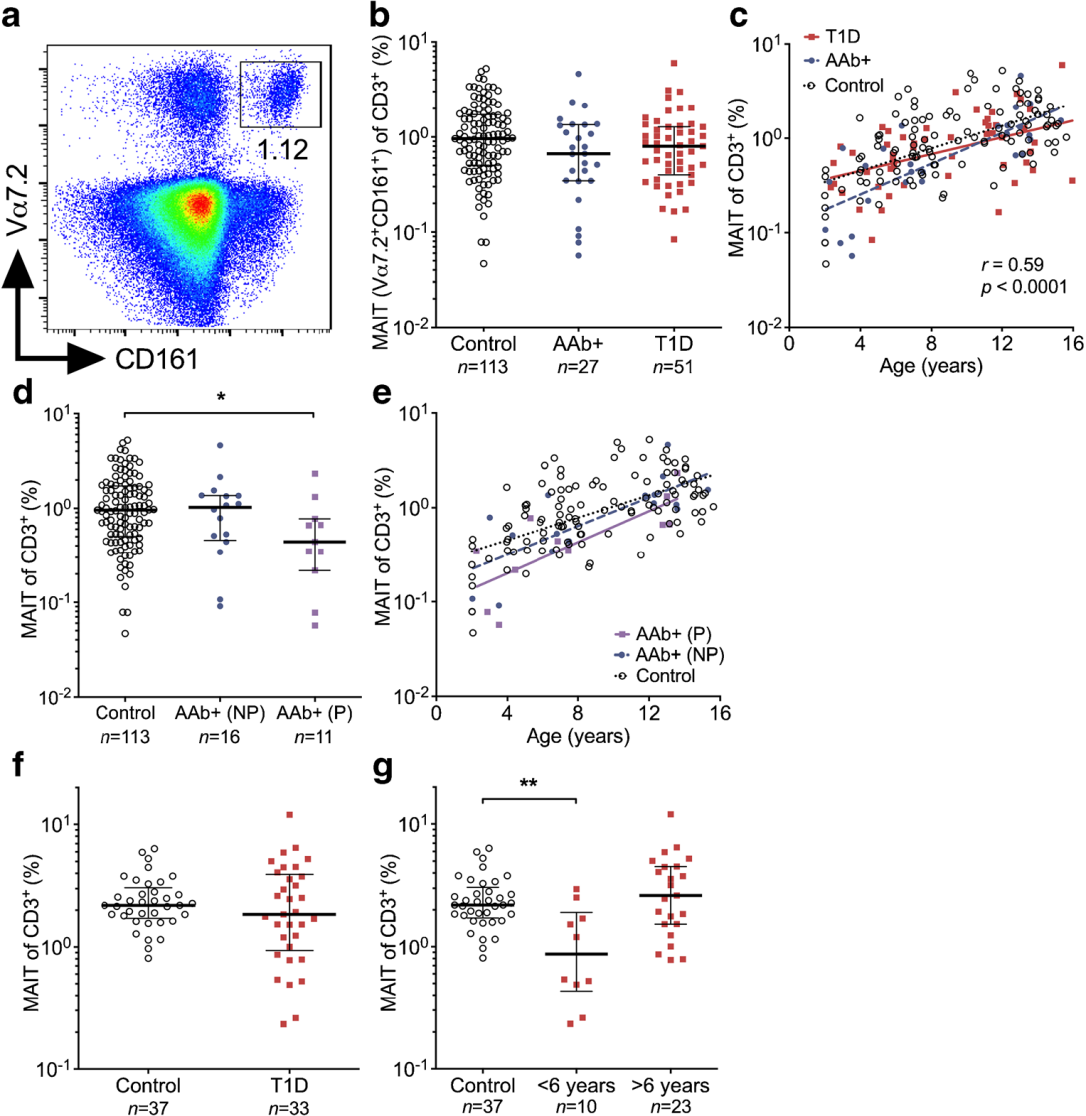
Decreased frequency of circulating MAIT cells in AAb^+^ children who progressed to type 1 diabetes (T1D) and in adult patients with a short duration of T1D. Representative example of flow cytometry gating of CD3^+^Vα7.2^+^CD161^+^ MAIT cells (**a**). The frequency of circulating MAIT cells in control, AAb^+^ and T1D groups in the paediatric cohort (**b**). Linear regression lines for log_10_-transformed MAIT cell frequencies vs age were calculated for the control, AAb^+^ and T1D groups (**c**). The slopes and elevations of the regression lines were not statistically different between the groups. Correlation with age was calculated by pooling all samples analysed and is expressed together with the *p* value on the plot. MAIT cell frequency in AAb^+^ children who did not progress (NP) or progressed (P) to T1D during the follow-up (**d**). Linear regression lines for log_10_-transformed MAIT cell frequencies vs age were calculated for the control, AAb^+^ non-progressor and AAb^+^ progressor groups (**e**). The elevations of the regression lines were statistically different among the groups (*p* < 0.05). Frequency of MAIT cells in adult healthy control individuals and adult patients with T1D (**f**) and in adult T1D patients with <6 years or >6 years since T1D diagnosis (**g**). (**b**, **d**, **f**, **g**) Median values with IQRs are shown, plotted on a log_10_ scale. *p < 0.05, **p < 0.01; Kruskal–Wallis test with Dunn’s post hoc test

We first analysed the frequency of circulating CD3^+^Vα7.2^+^CD161^+^ MAIT cells (Fig. 1a and ESM Fig. 1) in paediatric and adult cross-sectional cohorts (Table 1). In the paediatric cohort, the frequency of MAIT cells within the CD3^+^αβ^+^ T cell compartment was similar across the study groups (Fig. 1b). In accordance with previous reports [18, 21, 22], the frequency of MAIT cells demonstrated considerable interindividual variation and a strong positive correlation with the age of children (*r* = 0.59, *p* < 0.0001), with almost a tenfold increase in mean frequency between the ages of 2 and 15 years (Fig. 1c). However, even after stratification by age, we observed no differences in total MAIT cell frequencies between the study groups (Fig. 1c). Moreover, sex, HLA class II genotype, and blood glucose and HbA_1c_ levels did not appear to affect MAIT cell frequencies in the paediatric cohort (ESM Fig. 2). Although the frequency of MAIT cells was overall unaltered in AAb^+^ at-risk children (Fig. 1b), we interestingly observed that those AAb^+^ children who later progressed to type 1 diabetes had a decreased frequency of MAIT cells compared with control individuals (median 0.44% vs 0.96% of CD3^+^ T cells, *p* = 0.04; Fig. 1d). This result remained significant even after stratifying for age (Fig. 1e).

In the adult cohort, no differences in the frequency of MAIT cells were observed between adult patients with type 1 diabetes and healthy control individuals (Fig. 1f). Moreover, the frequency of MAIT cells in adults did not demonstrate correlation with age, sex, BMI or HbA_1c_ levels, or the duration of disease (ESM Fig. 2). However, we noted that some adult patients with a short disease duration had extremely low MAIT cell frequencies for adult individuals (<0.6% of CD3^+^ T cells; ESM Fig. 2). Consequently, when adult patients with diabetes were arbitrarily divided into two groups based on disease duration [<6 (mean 2.6) and >6 (mean 15.5) years after diagnosis], five of the ten patients with <6 years after diagnosis had a markedly lower frequency of MAIT cells compared with the 23 patients with >6 years after diagnosis or the 37 healthy control individuals (Fig. 1g). The median frequency of MAIT cells in patients with <6 years after diagnosis was 0.87% compared with 2.19% in healthy control individuals (*p* = 0.007; Fig. 1g).

In conclusion, no drastic changes in peripheral blood MAIT cell frequencies were observed in individuals with type 1 diabetes-associated autoimmunity. However, in subgroup analyses AAb^+^ at-risk children who later progressed to clinical diabetes as well as adult patients a short time after diagnosis displayed lower frequencies of circulating MAIT cells.

### Increased proportion of CD8^−^CD27^−^ MAIT cells in children with newly diagnosed type 1 diabetes

**Fig. 2:**
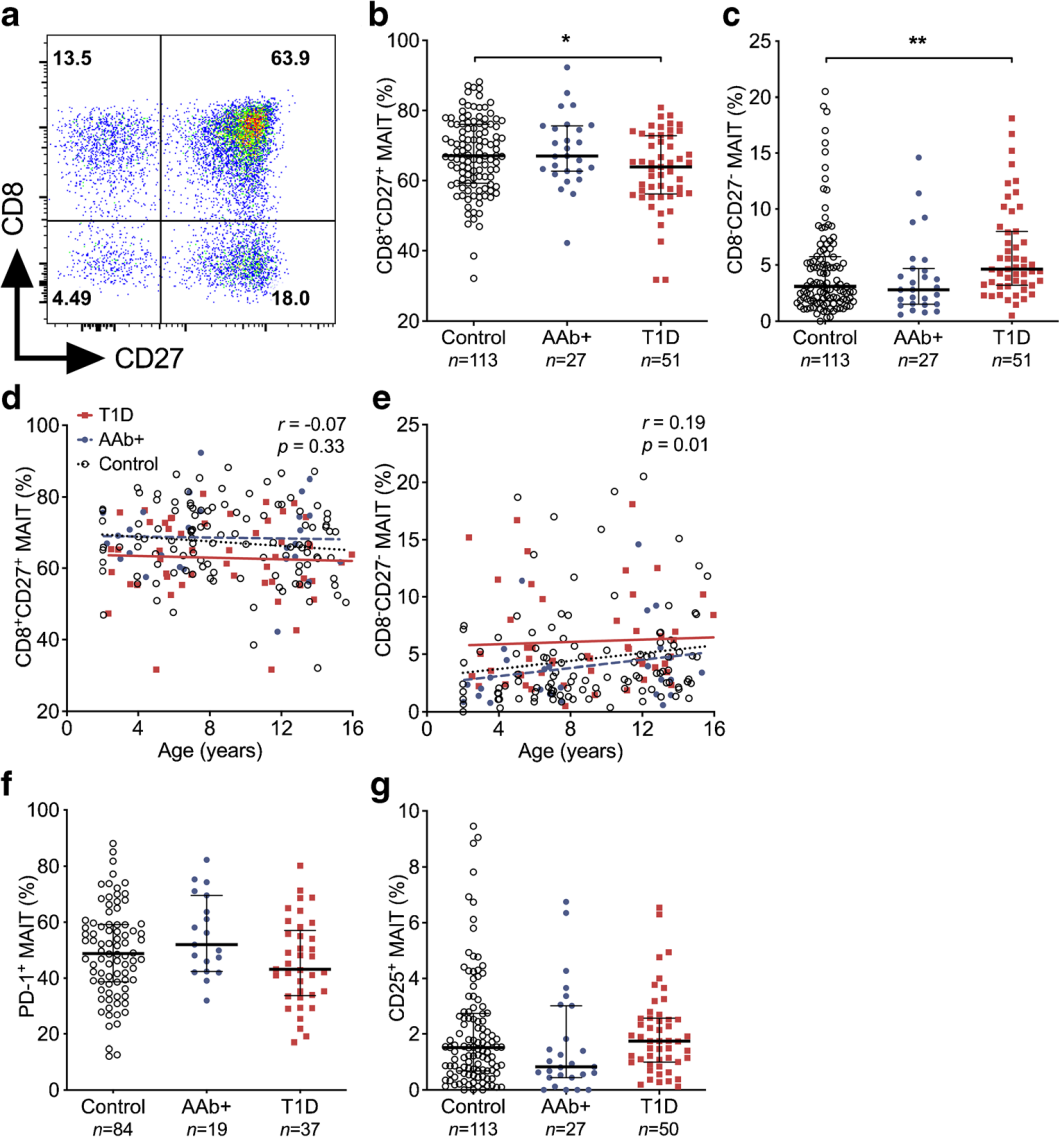
Proportional increase of CD8^−^CD27^−^ MAIT cells in children with newly diagnosed type 1 diabetes (T1D). Representative example of flow cytometry gating of CD8 and CD27 expression on MAIT cells (**a**). Proportions of CD8^+^CD27^+^ (**b**) and CD8^−^CD27^−^ (**c**) MAIT cells in control, AAb^+^ and T1D groups. Linear regression lines for CD8^+^CD27^+^ (**d**) and CD8^−^CD27^−^ (**e**) MAIT cell frequencies vs age were calculated for the control, AAb^+^ and T1D groups. The elevations of the regression lines were significantly different among the groups (*p* < 0.05). Correlation with age was calculated by pooling all samples analysed and is expressed together with *p* values on the individual plots. Proportions of PD-1^+^ (**f**) and CD25^+^ (**g**) MAIT cells in control, AAb^+^ and T1D groups. (**b**, **c**, **f**, **g**) Median values with IQRs are shown. *p < 0.05, **p < 0.01; Kruskal–Wallis test with Dunn’s post hoc test

We next analysed the frequencies of MAIT cell subsets expressing CD8 and/or CD27 (Fig. 2a). In the paediatric cohort, the proportion of CD8^+^CD27^+^ MAIT cells was decreased and that of CD8^−^CD27^−^ MAIT cells increased in children with type 1 diabetes compared with control children (median 63.9% vs 67.1% of MAIT cells, *p* = 0.04, and median 4.6% vs 3.1% of MAIT cells, *p* = 0.004, respectively; Fig. 2b, c), while the CD8^+^CD27^−^ and CD8^−^CD27^+^ MAIT cell subsets remained unaltered (ESM Fig. 3). These results remained significant even after stratifying for age (Fig. 2d, e). Importantly, no differences in conventional CD3^+^Vα7.2^+^CD161^−^ (non-MAIT), CD8^+^CD27^+^ or CD8^−^CD27^−^ T cell frequencies were observed between the groups (ESM Fig. 3), demonstrating that the observed alterations are specific to MAIT cells. No differences in the frequencies of CD8^+^CD27^+^ or CD8^−^CD27^−^ MAIT cells were observed in AAb^+^ children, even after stratification for progressors and nonprogressors, or in adult patients with type 1 diabetes (Fig. 2b, c and ESM Figs 3 and 4). There were also no differences in MAIT cells expressing the activation markers PD-1 and CD25 between the study groups in either the paediatric or adult cohorts (Fig. 2f, g and ESM Figs 3 and 4). In conclusion, we observed an increased proportion of CD8^−^CD27^−^ MAIT cells in children with newly diagnosed type 1 diabetes.

### Decreased CCR5, β7 integrin and IFN-γ expression on MAIT cells in children with newly diagnosed type 1 diabetes

**Fig. 3:**
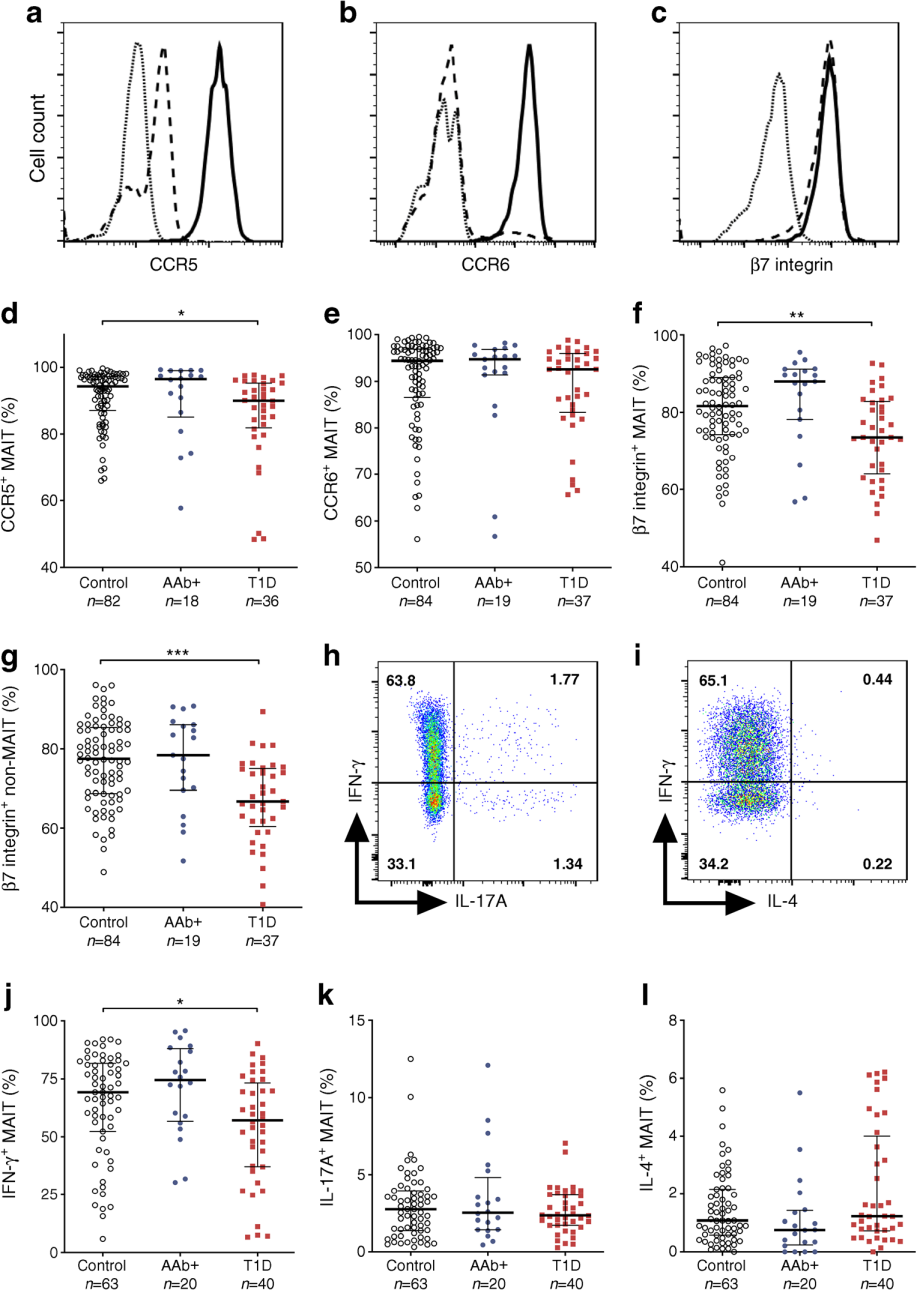
Lower expression of CCR5, β7 integrin and IFN-γ on MAIT cells from children with newly diagnosed type 1 diabetes (T1D). Representative examples of CCR5 (**a**), CCR6 (**b**) and β7 integrin (**c**) staining in fluorescence minus one (FMO) control (dotted lines), non-MAIT T cells (dashed lines) and MAIT cells (solid lines). Frequencies of CCR5^+^ (**d**), CCR6^+^ (**e**) and β7 integrin^+^ (**f**) MAIT cells as well as β7 integrin^+^ non-MAIT T cells (**g**) in control, AAb^+^ and T1D groups. Representative examples of IFN-γ, IL-17A and IL-4 production by MAIT cells (**h**, **i**). Frequencies of IFN-γ^+^ (**j**), IL-17A^+^ (**k**) and IL-4^+^ (**l**) MAIT cells in control, AAb^+^ and T1D groups. (**d**–**g**, **j**–**l**) Median values with IQRs are shown. *p < 0.05, **p < 0.01, ***p < 0.001; Kruskal–Wallis test with Dunn’s post hoc test

To better characterise the phenotype of MAIT cells during the progression to type 1 diabetes, we analysed the expression of homing receptors and cytokine production by MAIT cells in the paediatric cohort (ESM Fig. 1). MAIT cells express higher levels of CCR5 and CCR6 but similar levels of β7 integrin compared with non-MAIT T cells (Fig. 3a–c). We observed decreased levels of CCR5 and β7 integrin but not CCR6 expression on MAIT cells in children with type 1 diabetes compared with control children (median 90.0% vs 94.3% CCR5^+^ MAIT cells, *p* = 0.02, and median 73.5% vs 81.7% of β7 integrin^+^ MAIT cells, *p* = 0.004, respectively; Fig. 3d–f), even after stratification by age (ESM Fig. 5). The decrease in CCR5 expression appeared to be specific to MAIT cells since it was not altered in non-MAIT T cells (ESM Fig. 5). In contrast, β7 integrin expression was also decreased in non-MAIT T cells (as well as total CD3^+^ T cells) in children with type 1 diabetes compared with control children (median 66.8% vs 77.5% of non-MAIT T cells, *p* < 0.0001), suggesting a more global alteration of β7 integrin expression on T cells in children with type 1 diabetes (Fig. 3g and ESM Fig. 5). No changes in CCR5, CCR6 or β7 integrin expression were observed in AAb^+^ children (Fig. 3d–f and ESM Fig. 4).

To investigate the cytokine production profile of circulating MAIT cells, we stimulated PBMCs with PMA and ionomycin and analysed the production of IFN-γ, IL-17A and IL-4 (Fig. 3h, i). The frequency of IFN-γ^+^ MAIT cells was reduced in children with type 1 diabetes compared with control children (median 57.1% vs 69.3% of MAIT cells, *p* = 0.04; Fig. 3j). However, no differences in the frequencies of IL-17A^+^, IL-4^+^, IFN-γ^+^IL-17A^+^ or IFN-γ^+^IL-4^+^ MAIT cells were observed (Fig. 3k, l and ESM Fig. 5). The frequency of IFN-γ^+^ non-MAIT T cells was not altered (ESM Fig. 5). Once again, no differences in cytokine production were observed in AAb^+^ children (Fig. 3j–l and ESM Fig. 4). In conclusion, we observed that the expression of CCR5 and β7 integrin and the production of IFN-γ by MAIT cells were lower in children with newly diagnosed type 1 diabetes.

### Lower expression of β7 integrin and lower production of IFN-γ by CD8^−^CD27^−^ MAIT cells

**Fig. 4:**
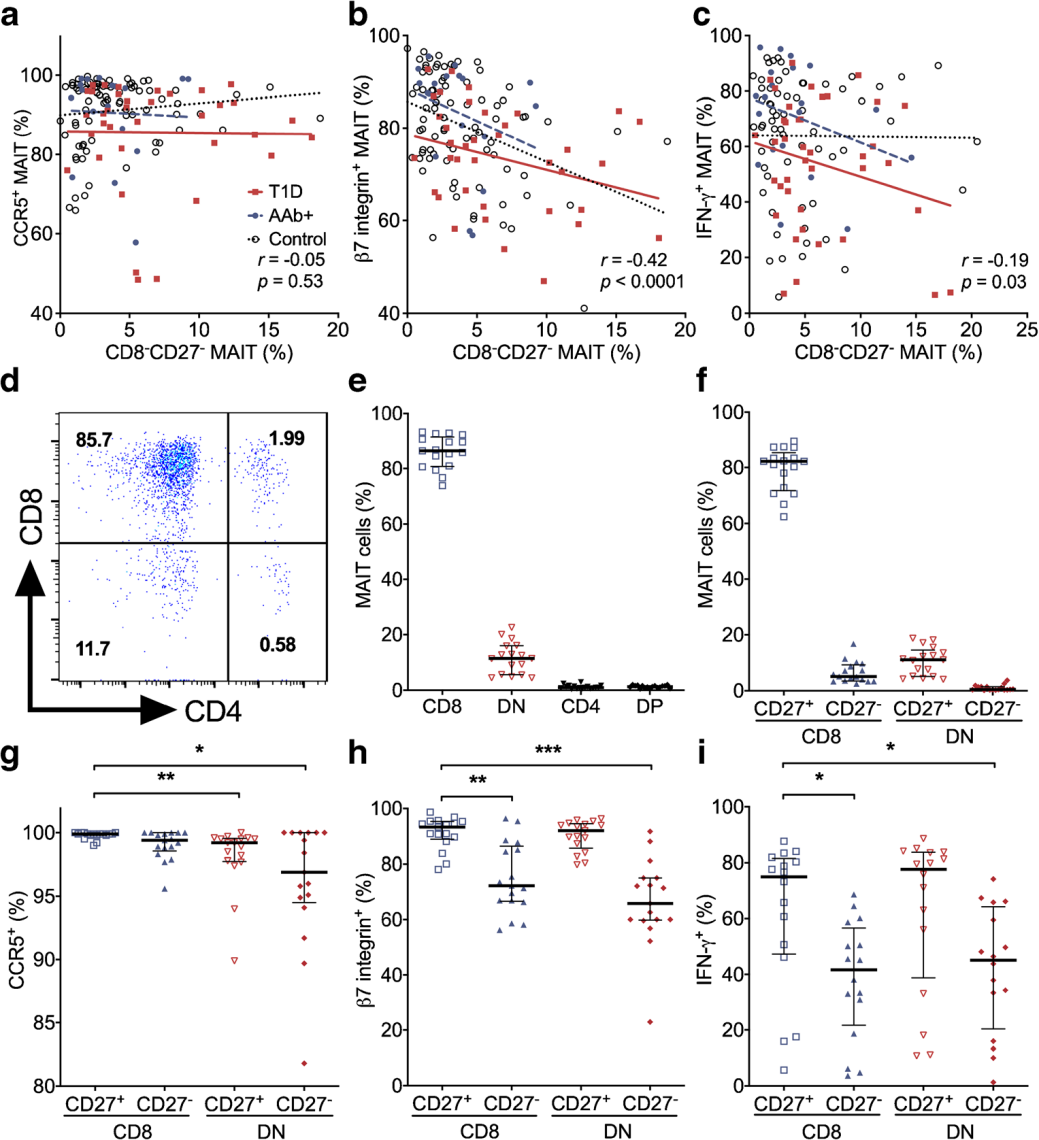
CCR5, β7 integrin and IFN-γ expression on CD8^−^CD27^−^ MAIT cells. Linear regression lines for CCR5^+^ (**a**), β7 integrin^+^ (**b**) and IFN-γ^+^ (**c**) MAIT cell frequencies vs CD8^−^CD27^−^ MAIT cell frequencies were calculated for the control, AAb^+^ and type 1 diabetes (T1D) groups. The slopes of the regression lines were not significantly different between the groups. Correlation with CD8^−^CD27^−^ MAIT cell frequencies was calculated by pooling all samples analysed and is expressed together with *p* values on the individual plots. Representative example of CD8 and CD4 expression on peripheral blood MAIT cells (**d**). The frequencies of MAIT cells that belong to the CD8^+^, DN, CD4^+^ and DP subsets (**e**), and that belong to the CD8^+^CD27^+^, CD8^+^CD27^−^, DN CD27^+^ and DN CD27^−^ subsets (**f**). Frequencies of CCR5^+^ (**g**), β7 integrin^+^ (**h**) and IFN-γ^+^ (**i**) cells within the MAIT cell subsets. The data in (**e**–**i**) represent a combined analysis of a total of 17 different healthy children. (**e**–**i**) Median values with IQRs are shown. *p < 0.05, **p < 0.01, ***p < 0.001; Kruskal–Wallis test with Dunn’s post hoc test

We next analysed whether the observed decrease in CCR5, β7 integrin and IFN-γ expression was mechanistically linked to the proportional increase in CD8^−^CD27^−^ MAIT cells in children with type 1 diabetes. The frequencies of β7 integrin^+^ and IFN-γ^+^ MAIT cells inversely correlated with the frequencies of CD8^−^CD27^−^ MAIT cells in the paediatric cohort, while the frequencies of CCR5^+^ MAIT cells did not demonstrate such correlation (Fig. 4a–c).

To study this further, we analysed additional paediatric healthy donors with a broader flow cytometry panel incorporating all the markers in the same analysis. In line with previous studies [21, 40, 41], we observed that around 80–90% of MAIT cells are CD8^+^ and almost all the remaining cells are double negative (DN), while CD4^+^ and double positive (DP) MAIT cells are extremely rare (Fig. 4d, e). Therefore, the CD8^−^CD27^−^ MAIT cells analysed so far are most likely to represent DN CD27^−^ cells. CD8^+^CD27^+^ MAIT cells represent about 80% of circulating MAIT cells followed by minor populations of DN CD27^+^, CD8^+^CD27^−^ and DN CD27^−^ MAIT cells (Fig. 4f). Next, we examined whether the expression of homing receptors and/or cytokines differs between the CD27-positive or -negative CD8^+^ and DN subsets. The expression of CCR5 was lower on DN CD27^−^ and DN CD27^+^ compared with CD8^+^CD27^+^ MAIT cells (Fig. 4g). Moreover, the frequencies of β7 integrin^+^ and IFN-γ^+^ MAIT cells were lower in CD8^+^CD27^−^ and DN CD27^−^ MAIT subsets compared with CD8^+^CD27^+^ MAIT cells (Fig. 4h, i). Of note, the frequencies of IL-4^+^ MAIT cells were also lower but those of IL-17A^+^ MAIT cells higher in CD8^+^CD27^−^ and DN CD27^−^ MAIT subsets compared with CD8^+^CD27^+^ MAIT cells (ESM Fig. 5).

Collectively, these analyses infer that the observed increase in CD8^−^CD27^−^ MAIT cells in children with type 1 diabetes (Fig. 2c) probably results from a proportional shift towards an increased frequency of DN CD27^−^ MAIT cells. This, in turn, explains the decreased β7 integrin expression and IFN-γ production by MAIT cells from children with type 1 diabetes (Fig. 3), as they demonstrate an inverse correlation with the frequency of CD8^−^CD27^−^ MAIT cells and their expression is markedly lower on DN CD27^−^ MAIT cells compared with CD8^+^CD27^+^ MAIT cells.

### MR1-dependent and cytokine-driven functional responses are not altered in MAIT cells from children with newly diagnosed type 1 diabetes or from AAb^+^ at-risk children

**Fig. 5:**
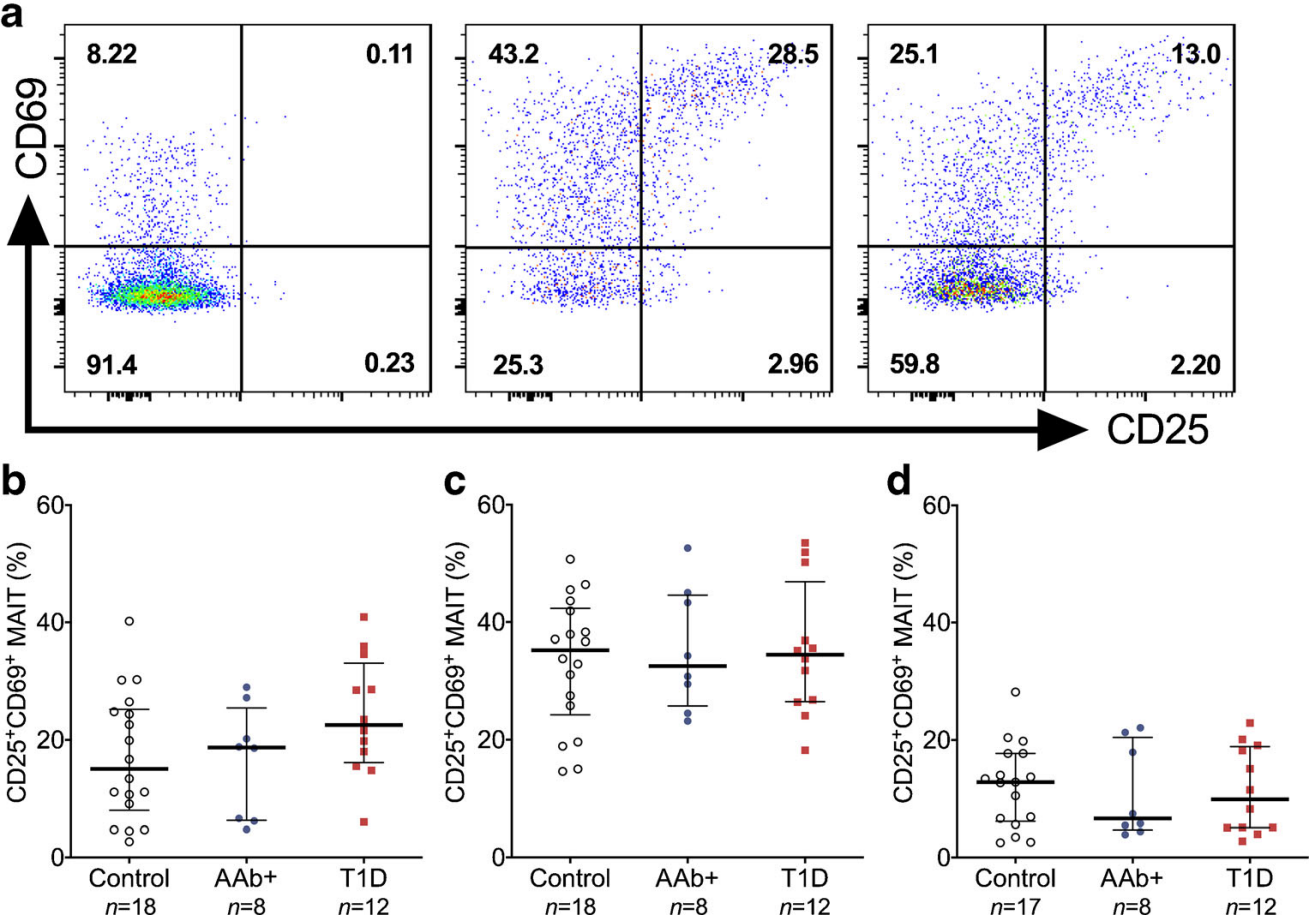
Comparable frequencies of CD25^+^CD69^+^ MAIT cells after stimulation in vitro with *E.coli* or the combination of IL-12 and IL-18 in children with type 1 diabetes (T1D), AAb^+^ children and control children. Representative example of CD25^+^CD69^+^ MAIT cells in an unstimulated PBMC sample (**a**, left), and samples stimulated at a PBMC to *E.coli* ratio 1:10 (**a**, middle) or with IL-12+IL-18 (**a**, right). Frequencies of CD25^+^CD69^+^ MAIT cells in samples stimulated at a PBMC to *E.coli* ratio 1:1 (**b**) and 1:10 (**c**), or with IL-12 and IL-18 (**d**) from paediatric control, AAb^+^ and T1D individuals. (**b**–**d**) Median values with IQRs are shown

To further investigate MAIT cell functionality during the progression of type 1 diabetes, PBMCs from 12 children with type 1 diabetes, nine AAb^+^ children and 18 control children were stimulated either with whole *E.coli* bacteria (MR1-dependent activation [39]) or with the combination of IL-12 and IL-18 (MR1-independent activation [42]). Activation of MAIT cells (CD69 and CD25 upregulation) was subsequently analysed (Fig. 5a). No differences in the frequencies of CD25^+^CD69^+^ MAIT cells were observed between the study groups upon stimulation with *E.coli* at two different concentrations (Fig. 5b, c) or with IL-12 and IL-18 (Fig. 5d). Moreover, no differences in the frequencies of CD25^+^CD69^+^ non-MAIT T cells were observed between the study groups (ESM Fig. 6). When anti-MR1 blocking antibodies were added to the *E.coli* stimulation, the frequencies of CD25^+^CD69^+^ MAIT cells but not non-MAIT cells were markedly reduced (ESM Fig. 6), confirming that *E.coli* activates MAIT cells through MR1 presentation. In conclusion, no impairment of MAIT cell activation was observed in children with type 1 diabetes or AAb^+^ at-risk children.

### Increased frequency of IL-17A-producing γδ T cells in children with newly diagnosed type 1 diabetes

**Fig. 6:**
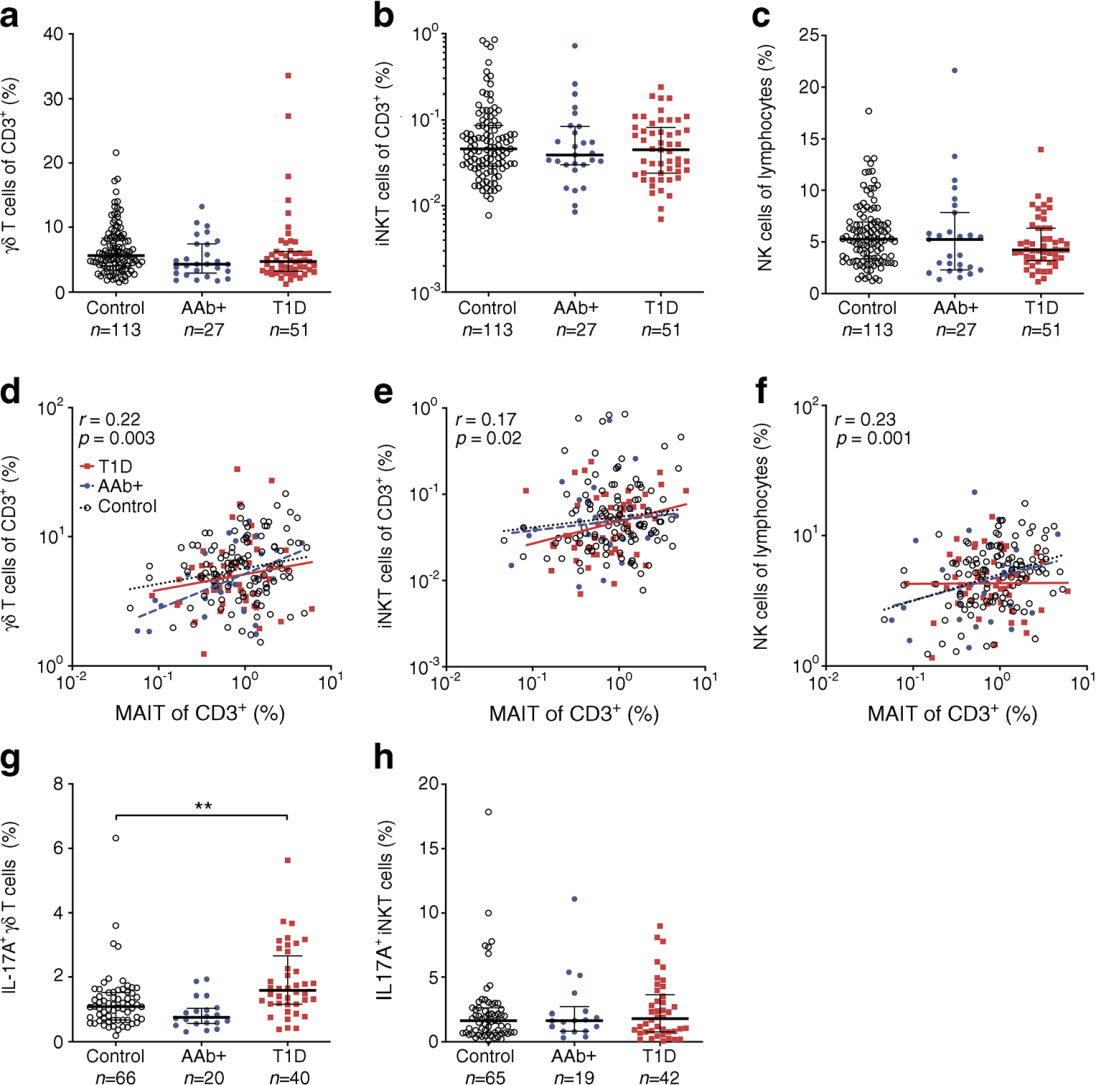
Increased frequency of IL-17A-producing γδ T cells in children with newly diagnosed type 1 diabetes (T1D). Frequencies of γδ T cells (**a**), iNKT cells (**b**) and NK cells (**c**) in control, AAb^+^ and T1D groups. Linear regression lines for log_10_-transformed γδ T cell (**d**), iNKT cell (**e**) and NK cell (**f**) frequencies vs log_10_-transformed MAIT cell frequencies were calculated for the control, AAb^+^ and T1D groups. The slopes and elevations of the regression lines were not statistically different between the groups. Correlation was calculated by pooling all samples analysed and is expressed together with *p* values on the individual plots. Frequencies of IL-17A-producing γδ T cells (**g**) and iNKT cells (**h**) in control, AAb^+^ and T1D groups. Median values with IQRs are shown; plotted on a log_10_ scale in (**b**).**p < 0.01; Kruskal–Wallis test with Dunn’s post hoc test

Finally, we analysed other peripheral blood UCT subsets, namely γδ T cells and iNKT cells, as well as natural killer (NK) cells in our paediatric cohort (ESM Figs 1 and 7). The frequencies of these cell types were similar between the study groups (Fig. 6a–c and ESM Fig. 4). Interestingly, the frequencies of γδ T cells, iNKT cells and NK cells appeared to correlate with circulating MAIT frequencies (Fig. 6d–f). However, in contrast to MAIT cells, the frequencies of γδ T cells or iNKT cells did not increase with age and NK cells displayed only a modest positive correlation with age (ESM Fig. 8).

To investigate whether there are phenotypic alterations in γδ T cells and iNKT cells, we analysed the production of IFN-γ, IL-17A and IL-4. No differences in the frequencies of IFN-γ^+^ and IL-4^+^ γδ T cells and iNKT cells were observed between the study groups (ESM Fig. 8). However, IL-17A^+^ γδ T cells but not iNKT cells were more abundant in children with newly diagnosed type 1 diabetes compared with control children (median 1.58% vs 1.09% of γδ T cells, *p* = 0.002; Fig. 6g and h and ESM Fig. 8). In conclusion, no changes in γδ T cells, iNKT cells and NK cells were observed in children with newly diagnosed type 1 diabetes, with the exception of an increased production of IL-17A by γδ T cells.

## Discussion

**Fig. 7:**
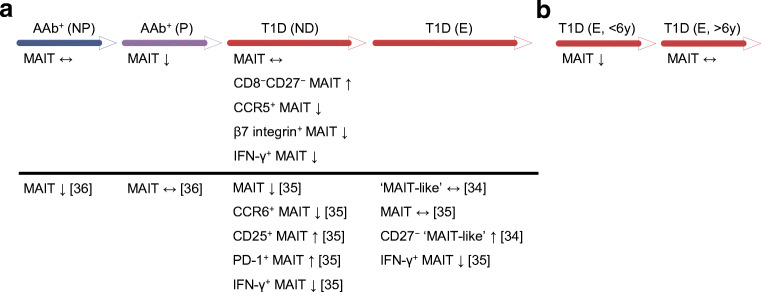
Peripheral blood MAIT cell alterations during the progression of type 1 diabetes (T1D). Alterations observed in our study in the paediatric cohort (**a**) and adult cohort (**b**) are displayed above the black line and those reported in previous studies [34–36] are listed below the black line. E, established (more than 10 days after diagnosis); ND, newly diagnosed (less than 10 days after diagnosis); NP, non-progressor; P, progressor

We observed multiple subtle alterations in the peripheral MAIT cell compartment through the analysis of cross-sectional cohorts of individuals at different stages of type 1 diabetes progression (Fig. 7). In children with newly diagnosed type 1 diabetes, the proportion of CD8^−^CD27^−^ (DN) MAIT cells was increased and the expression of the homing receptors CCR5 and β7 integrin, as well as the production of IFN-γ by MAIT cells was decreased. Moreover, the frequency of MAIT cells was decreased in AAb^+^ at-risk children who later progressed to type 1 diabetes and in adult patients with a short duration of type 1 diabetes. No alterations in MAIT cells were observed in AAb^+^ at-risk children who did not progress to diabetes during our follow-up or in adult type 1 diabetes patients with a long disease duration. These alterations were independent of age, which is a major factor affecting peripheral blood MAIT cell frequencies in children (Fig. 1c and [18, 21, 22]). Moreover, sex, HLA class II genotype, BMI or level of dysglycaemia were not observed to affect MAIT cell frequencies in our study cohorts (ESM Fig. 2). Consequently, our results suggest a temporal association between circulating MAIT cell alterations and the clinical onset of type 1 diabetes.

Of the MAIT cell alterations observed here, decreased expression of CD27 and production of IFN-γ have also been reported by others in patients with type 1 diabetes [34, 35] (Fig. 7). Some discrepancies between our and previous studies also remain (Fig. 7). Rouxel et al [35] reported a decreased frequency of total blood MAIT cells in children with type 1 diabetes, whereas this was not observed in our study. Also, the study by Harms et al did not detect differences in the frequency of ‘MAIT-like’ cells in children with type 1 diabetes [34]. A more recent study by the same group reported a decreased MAIT cell frequency in AAb^+^ at-risk individuals, especially in those who did not progress to type 1 diabetes [36], which contrasts with our finding of a decreased frequency of MAIT cells in AAb^+^ at-risk children who progressed to type 1 diabetes. Differences in gating strategy for MAIT cells [34], as well as how stringently the study cohorts have been matched for age and HLA background, may explain some of these discrepancies. Moreover, it is important to note that several additional factors that are difficult to control for, including previous infection history, the use of corticosteroids and antibiotics, as well as the presence of other inflammatory or metabolic diseases, can affect the circulating MAIT cell compartment considerably [43]. Due to the considerable interindividual variation in peripheral blood MAIT cell frequencies (Fig. 1c), subtle alterations may also be difficult to reproducibly detect with the cohort sizes analysed in studies performed thus far.

A clear (two- to fivefold) decrease in peripheral blood MAIT cell frequencies has consistently been observed in both patients with IBD [23–26] and SLE [27, 28]. In contrast, not all studies report decreased blood MAIT frequencies in patients with multiple sclerosis [31, 44] and rheumatoid arthritis [30], a finding that parallels the observations made in type 1 diabetes. Therefore, it is possible that alterations in blood MAIT cells may be less prominent in organ-specific autoimmune diseases (type 1 diabetes, multiple sclerosis and rheumatoid arthritis) compared with autoimmune diseases associated with a more systemic inflammatory response (IBD and SLE). In other autoimmune diseases, the decrease of blood MAIT cells has been attributed to either a preferential homing of the cells to inflamed target tissues [23, 25–27, 29, 33] or increased apoptosis caused by the inflammatory milieu [24, 28], which both may be less prominent in type 1 diabetes.

We provide evidence that all the observed alterations in MAIT cells in children with type 1 diabetes are likely mechanistically linked together. Compared with the most abundant CD8^+^CD27^+^ MAIT subpopulation, CD8^−^CD27^−^ (DN) MAIT cells express less CCR5 and β7 integrin and produce less IFN-γ (Fig. 4). Therefore, the proportional increase of CD8^−^CD27^−^ MAIT cells likely directly explains the reduced CCR5 and β7 integrin expression and IFN-γ production by circulating MAIT cells from children with type 1 diabetes. Alternatively, the decrease in the expression of the homing receptors CCR5 and β7 integrin could also be due to increased trafficking to inflamed tissues in children with type 1 diabetes, which would cause a proportional decrease in MAIT cells expressing these markers in the circulation. Interestingly, a proportional increase of blood (CD8^−^) DN MAIT cells has also been reported in patients with multiple sclerosis [31]. Recent research suggests that DN MAIT cells are a functionally mature effector subpopulation of MAIT cells that, at least in vitro, appear to derive from CD8^+^ MAIT cells under chronic TCR stimulation [45]. Less is known about the relevance of CD27 on MAIT cell functionality. However, at least in conventional T cells the loss of CD27 is associated with a more terminal effector phenotype of T cells [46]. Collectively, our results therefore suggest a shift towards a more terminally differentiated MAIT phenotype in blood at the time of type 1 diabetes diagnosis.

One caveat of our study is that we could only analyse cross-sectional cohorts and further work on longitudinal sample sets would be required to confirm the temporal association of peripheral blood MAIT cell alterations and the onset of type 1 diabetes. Another obvious caveat of our study is that we could only analyse MAIT cells in blood samples, where immune changes are likely to be minor compared with those in inflamed islets. In the NOD mouse model, MAIT cells accumulate in pancreatic islets during disease progression and increase their expression of IFN-γ and granzyme B [35]. However, in the absence of MAIT cells, there is an accelerated progression to diabetes in NOD mice, suggesting that MAIT cells may actually have a protective role in type 1 diabetes, possibly due to their role in supporting gut integrity [35]. Importantly, a recent study showed that MAIT cells were completely absent in human insulitic lesions in pancreas biopsies obtained from six patients with newly diagnosed type 1 diabetes [47]. Collectively, these studies support the notion that MAIT cells may not play a direct role in the beta cell destructive process in human type 1 diabetes, but that their peripheral blood alterations rather reflect a response to the inflammatory changes during diabetes progression.

The frequencies of other circulating UCT subtypes, namely γδ T cells and iNKT cells, were not altered either in children with newly diagnosed type 1 diabetes or AAb^+^ at-risk children. However, in line with previous reports [21, 22, 32], the frequencies of these cell subtypes correlated with MAIT cell frequencies in blood, suggesting that MAIT cells, γδ T cells and iNKT cells may share common developmental and/or homeostatic factors. In contrast to earlier reports [48, 49], we did not observe a reduced frequency of NK cells in children with type 1 diabetes. No changes in the cytokine production patterns of γδ T cells and iNKT cells were observed, except for an increased frequency of IL-17A^+^ γδ T cells in children with type 1 diabetes. Interestingly, IL-17A^+^ γδ T cells have been demonstrated to be an important pathogenic effector cell subset in the NOD mouse model [50]. Therefore, our current observation may warrant further investigation on the role of IL-17A-producing γδ T cells in human type 1 diabetes. Of note, in addition to MAIT cells we also observed decreased expression of β7 integrin on conventional CD3^+^ T cells in children with type 1 diabetes (Fig. 3), which to our knowledge has not been reported previously. This finding would suggest a more global defect in mucosal T cell homing in children with type 1 diabetes, which should also be explored in more detail in the future.

In conclusion, we present here a thorough analysis of peripheral blood MAIT cells during human type 1 diabetes development, utilising the largest and most stringently age-matched cohorts analysed so far, to our knowledge. We were able to demonstrate subtle alterations in circulating MAIT cells, suggesting a shift towards a more terminally differentiated MAIT phenotype in children with newly diagnosed type 1 diabetes. We also observed a decreased frequency of MAIT cells in AAb^+^ children who later progressed to type 1 diabetes as well as in adult type 1 diabetes patients with a short disease duration. Taken together, our current data suggest that peripheral blood MAIT cell alterations are temporally associated with the clinical onset of type 1 diabetes.

## Electronic supplementary material

ESM (PDF 1565 kb)

## Data Availability

The datasets generated during the current study are available from the corresponding author upon reasonable request.

## Abbreviations

AAb^+^: Autoantibody-positive
CCR: C-C chemokine receptor
DN: Double negative
DP: Double positive
IBD: Inflammatory bowel disease
iNKT: Invariant natural killer T (cell)
MAIT: Mucosal-associated invariant T (cell)
MR1: MHC-Ib-related protein 1
NK: Natural killer
PBMC: Peripheral blood mononuclear cell
PD-1: Programmed cell death protein 1
PMA: Phorbol myristic acid
SLE: Systemic lupus erythematosus
TCR: T cell receptor
UCT cell: Unconventional T cell
