# Supplementary material for: Mucosal-associated invariant T cell alterations during the development of human type 1 diabetes
Source: Diabetologia. 2020 Sep 3;63(11):2396–409. doi: 10.1007/s00125-020-05257-7 (PMC7527319; doi:10.1007/s00125-020-05257-7)
Supplement: Supplementary file 1 — (PDF 1565 kb) [file 125_2020_5257_MOESM1_ESM.pdf]

ESM Table 1 Fluorescent monoclonal antibodies used in the study

Staining panel	Antibody	Manufacturer	Clone
**MAIT core panel (used in panels 1-6)**	Zombie aqua (Live/dead)	BioLegend	N/A
	CD3 APC-F750	BioLegend	SK7
	TCR Vα7.2 BV421	BioLegend	3C10
	CD161 PerCP-Cy5.5	BioLegend	HP-3G10
**MAIT panel 1 (Figures 1, 2 and 6; FACSCanto II)**	TCR γδ PE-Cy7	BioLegend	B1
	CD8 A488	BioLegend	RPA-T8
	CD25 PE	Miltenyi Biotec	4E3
	CD27 A647	BioLegend	O323
**MAIT panel 2 (Figures 2 and 3; FACSCanto II)**	CCR6 PE-Cy7	BioLegend	G034F3
	CCR5 A488	BioLegend	J418F1
	PD-1 PE	BioLegend	EH12.2H7
	β7 integrin A647	BioLegend	FIB504
**MAIT panel 3 (Figure 3; FACSCanto II)**	TCR γδ PE-Cy7	BioLegend	B1
	IFN-γ A488	BioLegend	4S.B3
	IL-17A PE	BioLegend	BL168
	IL-4 A647	BioLegend	8D4-8
**MAIT panel 4 (Figure 4; Cytoflex S)**	CD4 PE-Cy7	BioLegend	RPA-T4
	IFN-γ A488	BioLegend	4S.B3
	IL-17A PE	BioLegend	BL168
	IL-4 A647	BioLegend	8D4-8
	CD8 BV605	BioLegend	RPA-T8
	CD27 APC-R700	BD Bioscience	M-T271
**MAIT panel 5 (Figure 4; Cytoflex S)**	CD4 PE-Cy7	BioLegend	RPA-T4
	CCR5 A488	BioLegend	J418F1
	CCR6 PE	BioLegend	G034F3
	β7 integrin A647	BioLegend	FIB504
	CD8 BV605	BioLegend	RPA-T8
	CD27 APC-R700	BD Bioscience	M-T271
**MAIT panel 6 (Figure 5; FACSCanto II)**	TCR γδ PE-Cy7	BioLegend	B1
	CD69 FITC	BioLegend	FN50
	CD25 PE	Miltenyi Biotec	4E3

ESM Table 1 (continued)

**iNKT panel 1 (Figure 6; FACSCanto II)**	Zombie aqua (Live/dead)	BioLegend	N/A
	CD3 APC-F750	BioLegend	SK4
	TCR Vα24-J18 BV421	BioLegend	6B11
	CD56 PerCp-Cy5.5	BioLegend	HCD56
	CD4 PE-Cy7	BioLegend	RPA-T4
	CD16 A488	BioLegend	3G8
	CD25 PE	Miltenyi Biotec	4E3
	CD27 A647	BioLegend	O323
**iNKT panel 2 (Figure 6; FACSCanto II)**	Zombie aqua (Live/dead)	BioLegend	N/A
	CD3 APC-F750	BioLegend	SK4
	TCR Vα24-J18 BV421	BioLegend	6B11
	CD45RA PerCp-Cy5.5	BioLegend	HI100
	CD4 PE-Cy7	BioLegend	RPA-T4
	IFN-γ A488	BioLegend	4S.B3
	IL-17A PE	BioLegend	BL168
	IL-4 A647	BioLegend	8D4-8

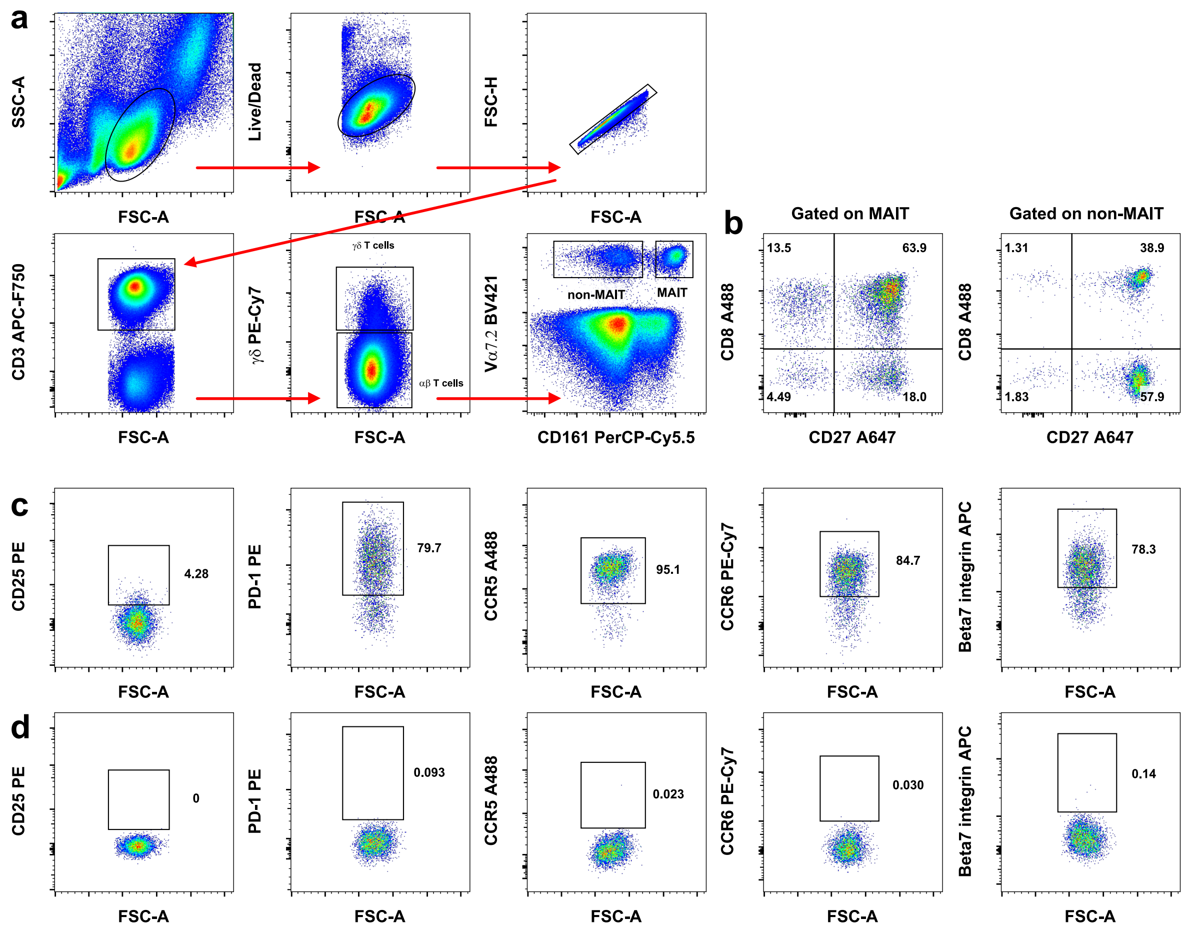

ESM Figure 1 (**a**) Gating strategy to define circulating MAIT cells. Lymphocytes were first gated based on FSC and SSC properties, followed by exclusion of dead cells and elimination of doublets through FSC-A and FSC-H gating. MAIT cells were gated as $CD3^{+}\gamma\delta^{-}V\alpha7.2^{+}CD161^{+}$, conventional non-MAIT T cells as $CD3^{+}\gamma\delta^{-}V\alpha7.2^{+}CD161^{-}$ and γδ T cells as $CD3^{+}\gamma\delta^{+}$. Representative examples of CD8/CD27 subpopulations within MAIT and non-MAIT cells (**b**). Representative examples of CD25, PD-1, CCR5, CCR6 and β7 integrin expression on MAIT cells (**c**) together with the corresponding fluorescence minus one (FMO) control (**d**).

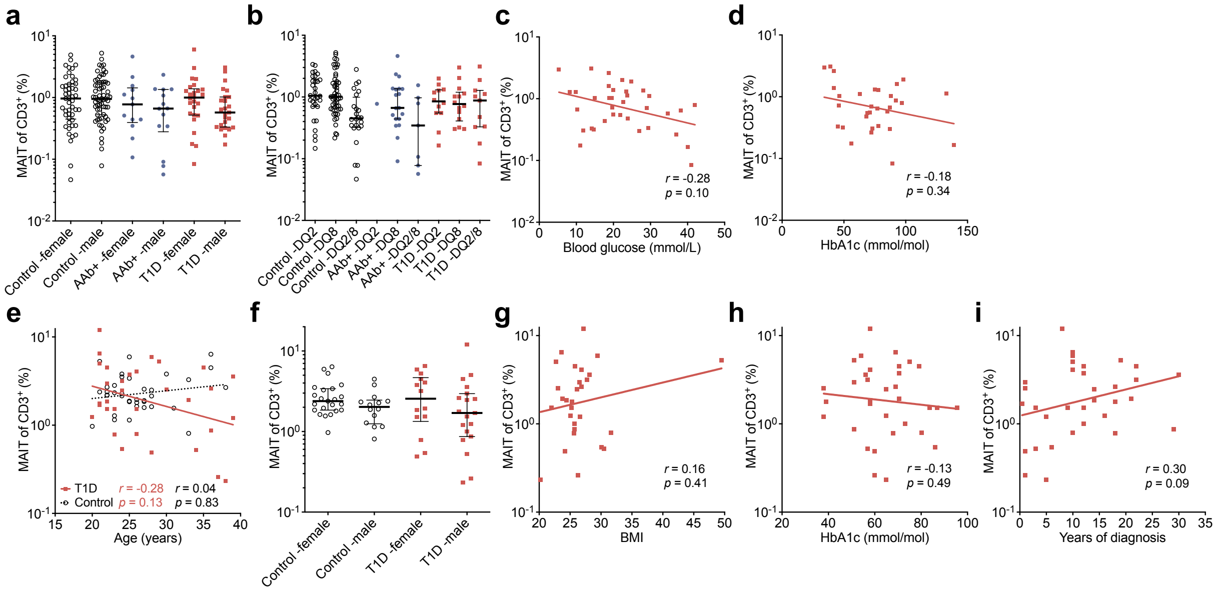

ESM Figure 2 Effect of different clinical parameters on MAIT cell frequencies. The effect of sex (**a**) and HLA class II genotype (**b**) on MAIT cell frequencies in the paediatric cohort. Linear regression for $\log_{10}$-transformed MAIT cell frequencies against random blood glucose values (**c**) and HbA1c (**d**) levels at diagnosis in children with newly diagnosed type 1 diabetes. Linear regression lines for $\log_{10}$-transformed MAIT cell frequencies against age were calculated for the adult control (dotted, black line) and adult T1D (solid, red lines) groups (**e**). The effect of sex on MAIT cell frequencies in the adult cohort (**f**). Linear regression for $\log_{10}$-transformed MAIT cell frequencies against body mass index (BMI; **g**), HbA1c levels (**h**) and years from T1D diagnosis (**i**) in adult T1D patients. Correlations with *p* values are shown in (**c-e** and **g-i**).

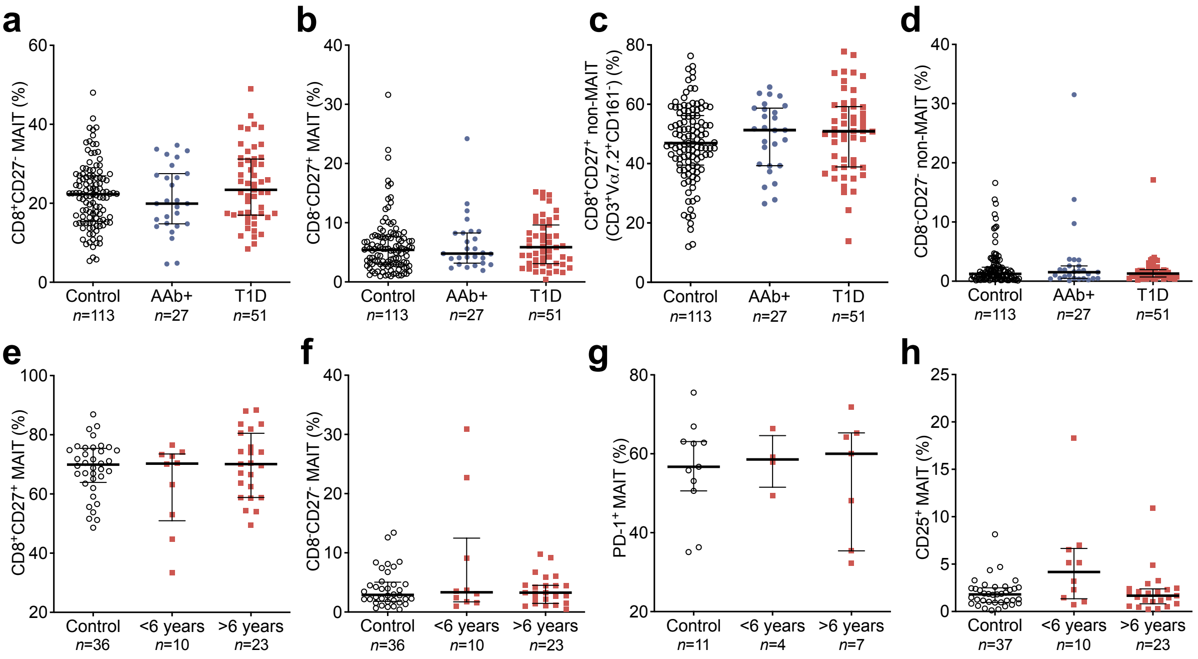

ESM Figure 3 Analysis of CD8, CD27, PD-1 and CD25 expression within circulating MAIT cells. Proportions of $CD8^{+}CD27^{-}$ (**a**) and $CD8^{-}CD27^{+}$ (**b**) MAIT cells in paediatric control, AAb^{+} and type 1 diabetes (T1D) groups. Proportions of $CD8^{+}CD27^{+}$ (**c**) and $CD8^{-}CD27^{-}$ (**d**) non-MAIT T cells in control, AAb^{+} and T1D groups. Proportions of $CD8^{+}CD27^{+}$ (**e**), $CD8^{-}CD27^{-}$ (**f**), $PD\text{-}1^{+}$ (**g**) and $CD25^{+}$ (**h**) MAIT cells in adult healthy controls and adult T1D patients with less than 6 years or more than 6 years since T1D diagnosis. Median values with interquartile ranges are shown.

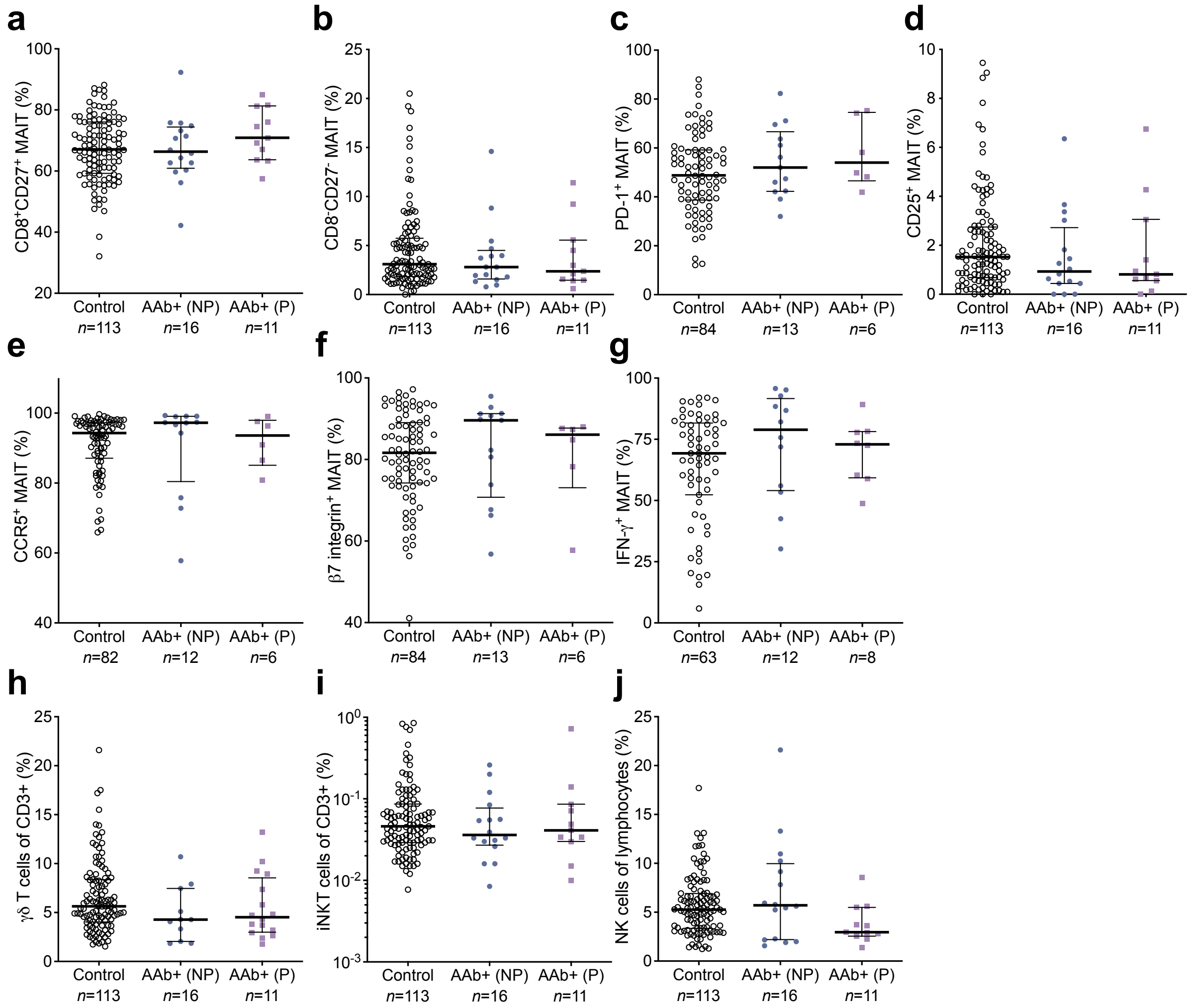

ESM Figure 4 Analysis of circulating MAIT cells in AAb$^+$ at-risk children. AAb$^+$ children were divided into non-progressors (NP) and progressors (P). The proportion of CD8$^+$CD27$^+$ (**a**), CD8$^-$CD27$^-$ (**b**), PD-1$^+$ (**c**), CD25$^+$ (**d**), CCR5+ (**e**), β7 integrin+ (**f**) and IFN-γ$^+$ (**g**) MAIT cells in control, non-progressor and progressor groups. Frequencies of γδ T cells (**h**), iNKT cells (**i**) and NK cells (**j**) in control, non-progressor and progressor groups. Median values with interquartile ranges are shown.

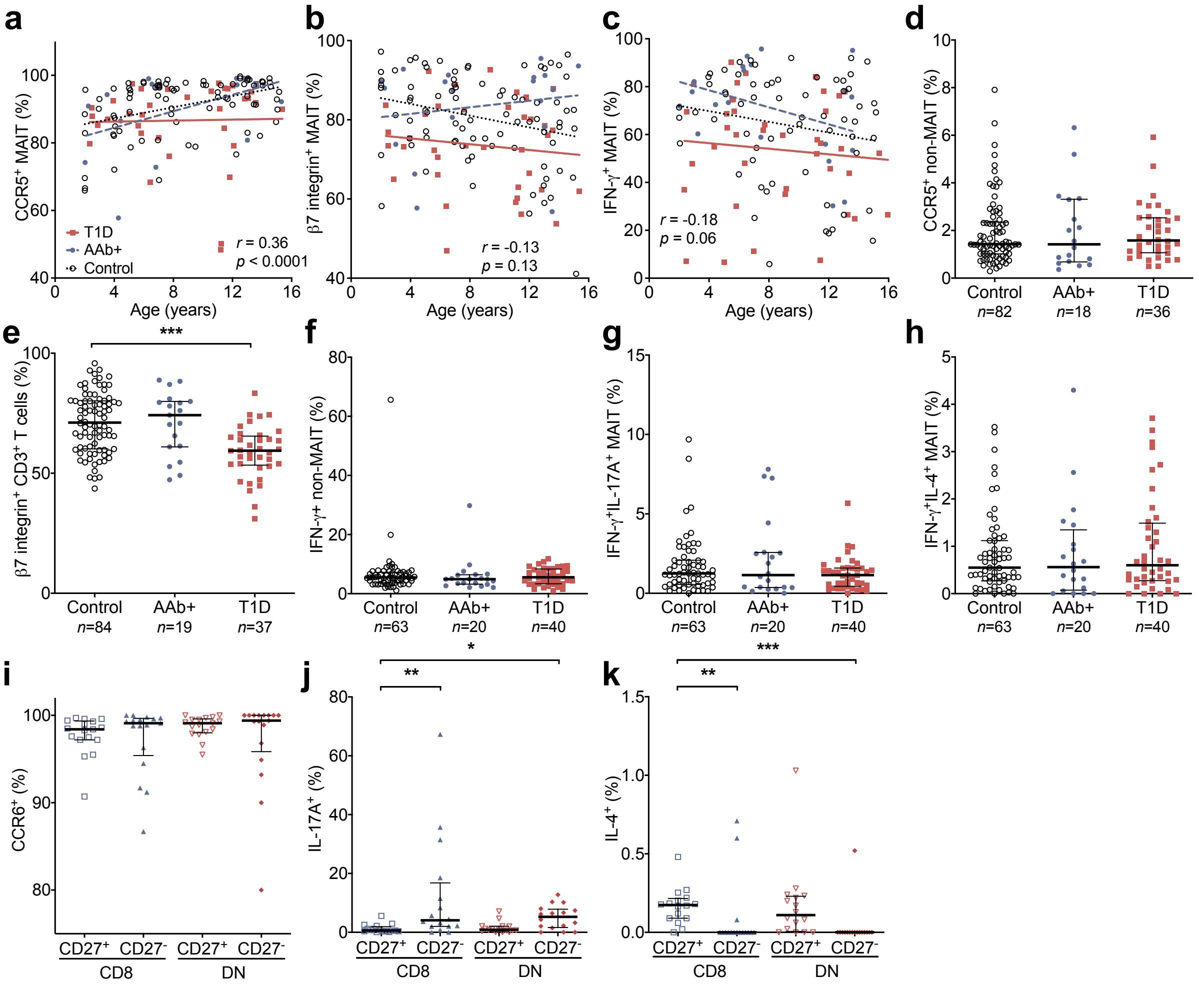

ESM Figure 5 Analysis of homing receptor expression and cytokine production on MAIT cells in the paediatric cohort. Linear regression lines for CCR5$^+$ (**a**), β7 integrin$^+$ (**b**), and IFN-γ$^+$ (**c**) MAIT cell frequencies against age were calculated for the control (dotted, black lines), AAb$^+$ (dashed, blue lines) and type 1 diabetes (T1D; solid, red lines) groups. The elevations of the regression lines were significantly different between the groups for CCR5$^+$, β7 integrin$^+$, and IFN-γ$^+$ MAIT cells (p<0.05). Correlation with age was calculated by pooling all samples analysed and is expressed together with p values next to the individual plots. Frequencies of CCR5$^+$ non-MAIT T cells (**d**), β7 integrin$^+$ total CD3$^+$ T cells (**e**) and IFN-γ$^+$ non-MAIT T cells (**f**), as well as IFN-γ$^+$IL-17A$^+$ (**g**) and IFN-γ$^+$IL-4$^+$ (**h**) MAIT cells in control, AAb$^+$ and T1D groups. Frequencies of CCR6$^+$ (**i**), IL-17A$^+$ (**j**) and IL-4$^+$ (**k**) cells within MAIT cell subsets defined by CD8 and CD27 expression, representing a combined analysis of a total of 17 different healthy children. Median values with interquartile ranges are shown. *p<0.05, **p<0.01, ***p<0.001; Kruskal-Wallis test with Dunn's post hoc test.

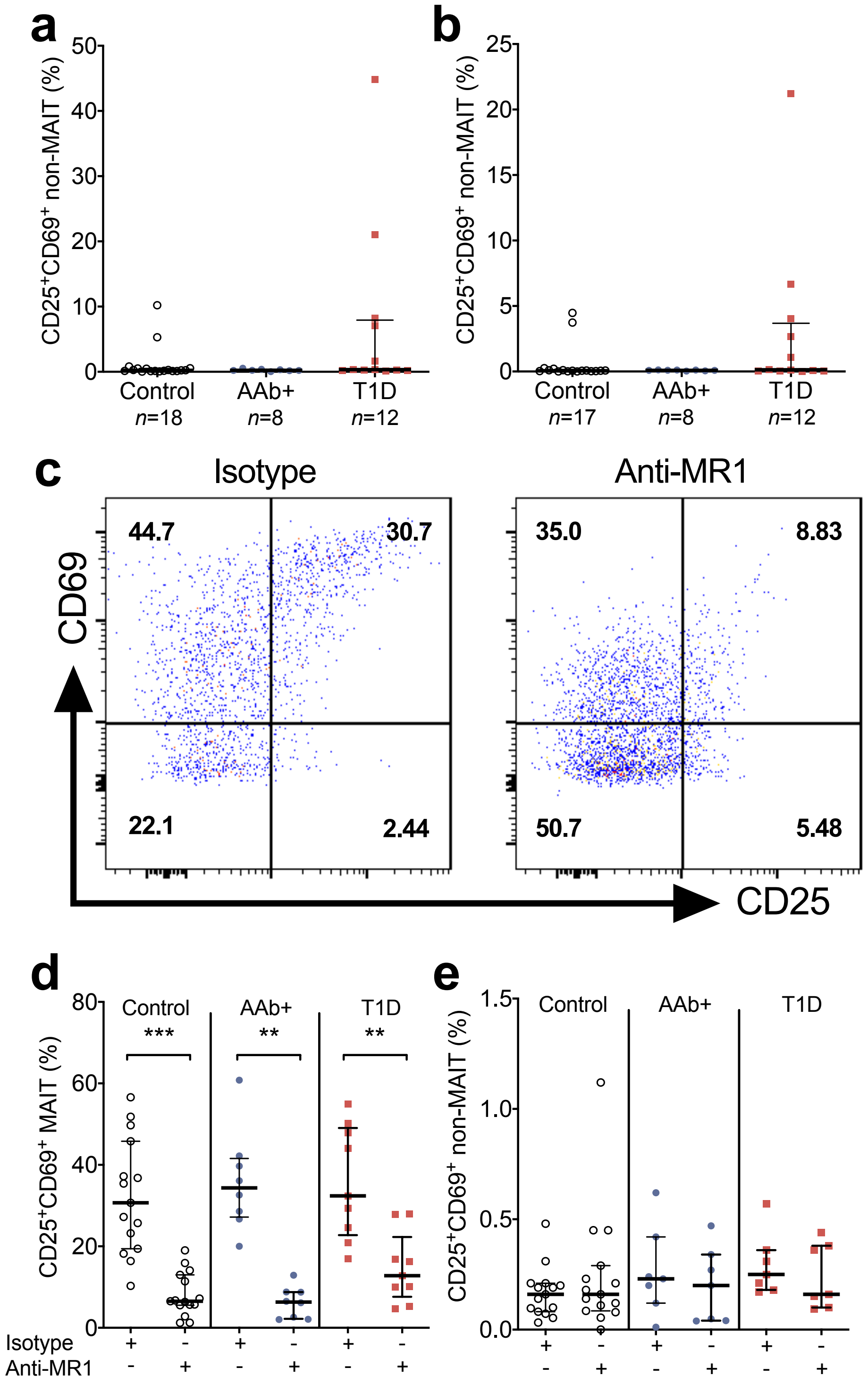

ESM Figure 6 Functional assays for MAIT cell activation *in vitro*. Frequencies of $CD25^{+}CD69^{+}$ non-MAIT T cells in samples stimulated at a PBMC to *E.coli* ratio 1:10 (**a**) or with IL-12 and IL-18 (**b**) from paediatric control, AAb^{+} and type 1 diabetes (T1D) individuals. Representative example of PBMC samples stimulated at a PBMC to *E.coli* ratio 1:10 together with either an isotype control or anti-MR1 blocking antibody (**c**). Frequencies of $CD25^{+}CD69^{+}$ MAIT cells (**d**) and non-MAIT T cells (**e**) in cultures stimulated at a PBMC to *E.coli* ratio 1:10 together with isotype control or anti-MR1 blocking antibodies. Median values with interquartile ranges are shown. ${**}p<0.01$, ${***}p<0.001$; Mann-Whitney U-test.

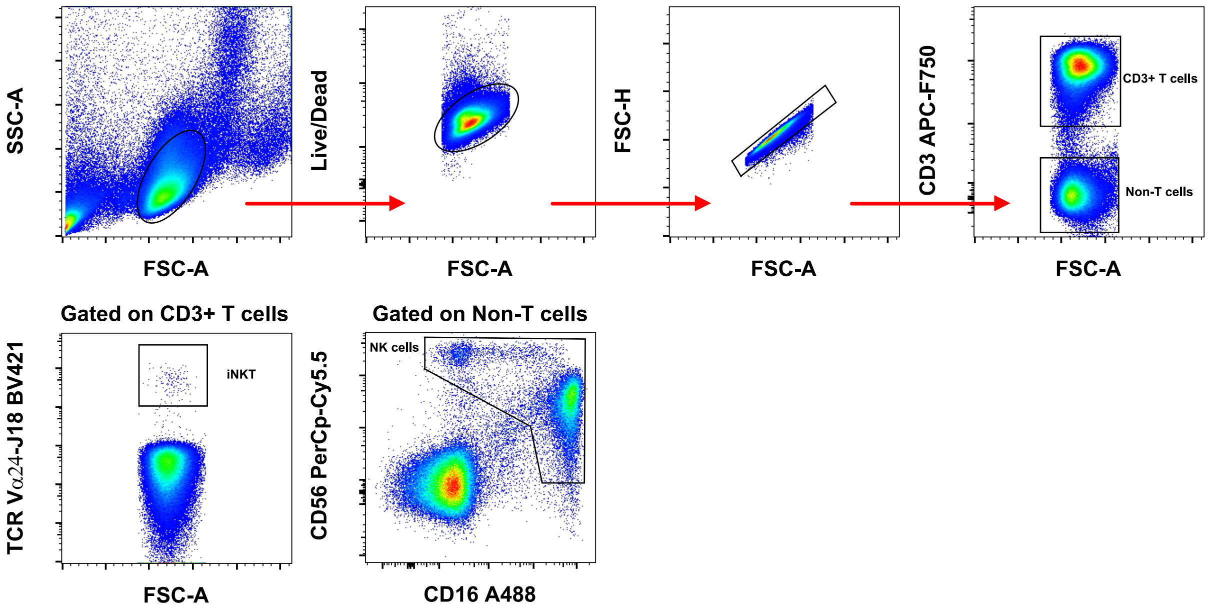

ESM Figure 7 Gating strategies to define circulating iNKT and NK cells. Lymphocytes were first gated based on FSC and SSC properties, followed by exclusion of dead cells and elimination of doublets through FSC-A and FSC-H gating. iNKT cells were gated as $CD3^{+}V\alpha24\text{-}J18^{+}$ and NK cells as $CD3^{-}CD56^{+}CD16^{+/-}$.

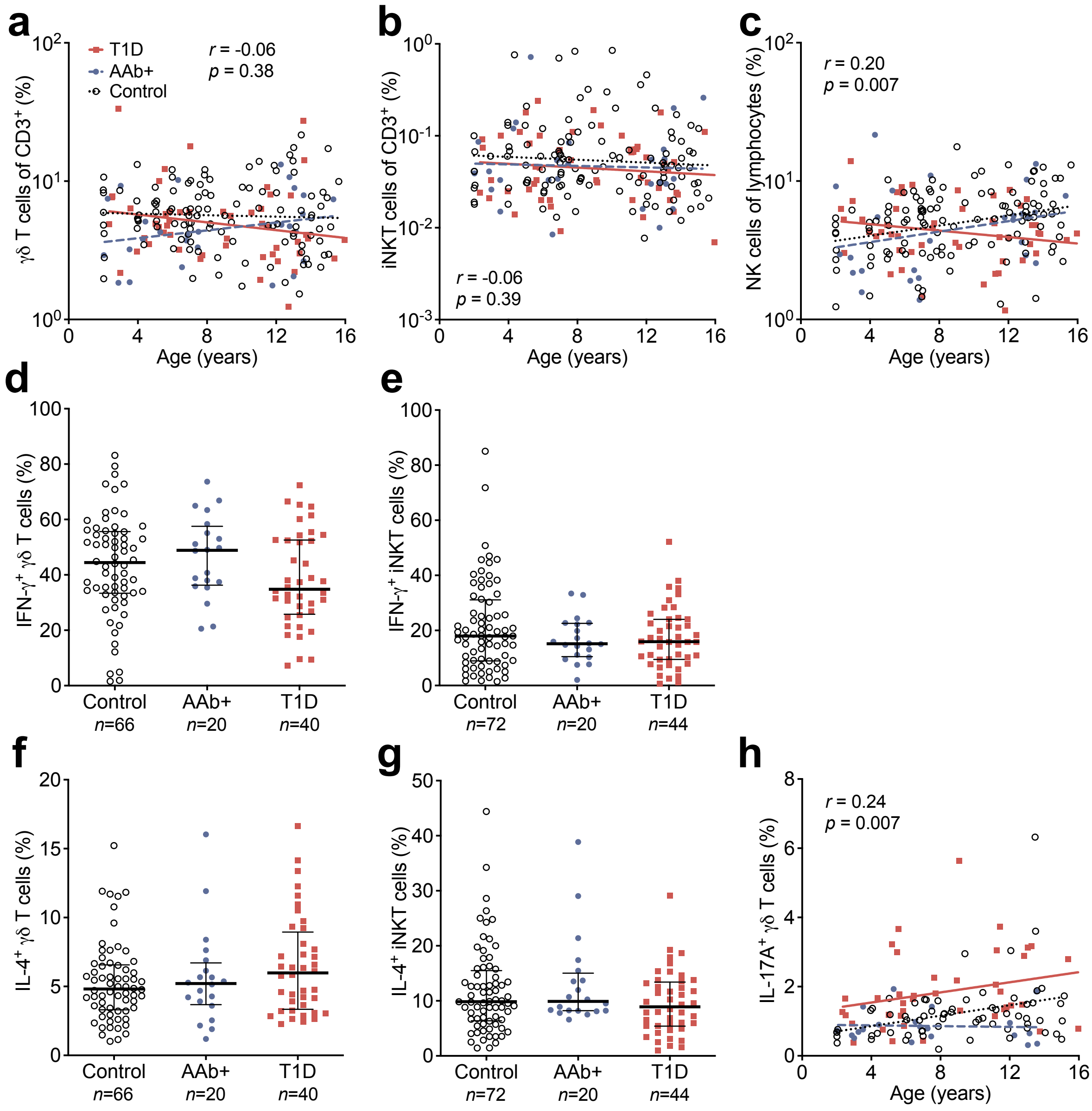

ESM Figure 8 Analysis of circulating unconventional T cell subsets in the paediatric cohort. Linear regression lines for $\log_{10}$-transformed γδ T cells (**a**), iNKT cells (**b**) and NK cells (**c**) against age were calculated for the control (dotted, black lines), AAb^+ (dashed, blue lines) and type 1 diabetes (T1D; solid, red lines) groups. The slopes and elevations of the regression lines were not statistically different between the groups. Correlation with age was calculated by pooling all samples analysed and is expressed together with *p* values next to the individual plots. Frequencies of IFN-γ^+ and IL-4^+ γδ T cells and iNKT cells in control, AAb^+ and T1D groups (**d-g**). Linear regression lines for IL-$17A^+$ γδ T cells against age were calculated for the control (dotted, black lines), AAb^+ (dashed, blue lines) and T1D (solid, red lines) groups (**h**). The elevations of the regression lines were statistically different between the groups ($p<0.05$). Correlation with age was calculated by pooling samples analysed and is expressed together with *p* value. Median values with interquartile ranges are shown.
